# Cortico-Hippocampal Computational Modeling Using Quantum-Inspired Neural Networks

**DOI:** 10.3389/fncom.2020.00080

**Published:** 2020-11-05

**Authors:** Mustafa Khalid, Jun Wu, Taghreed M. Ali, Thaair Ameen, Ali Salem Altaher, Ahmed A. Moustafa, Qiuguo Zhu, Rong Xiong

**Affiliations:** ^1^State Key Laboratory of Industrial Control Technology, Institute of Cyber-Systems and Control, Zhejiang University, Hangzhou, China; ^2^Binhai Industrial Technology Research Institute of Zhejiang University, Tianjin, China; ^3^Electrical Engineering Department, University of Baghdad, Baghdad, Iraq; ^4^Institute of Computer Science, Zhejiang University, Hangzhou, China; ^5^CEECS Department, Florida Atlantic University, Boca Raton, FL, United States; ^6^Marcs Institute for Brain and Behaviour and School of Psychology, Western Sydney University, Sydney, NSW, Australia; ^7^Department of Human Anatomy and Physiology, Faculty of Health Sciences, University of Johannesburg, Johannesburg, South Africa

**Keywords:** quantum-inspired neural network, computational modeling, classical conditioning, learning algorithm, cortico-hippocampal

## Abstract

Many current computational models that aim to simulate cortical and hippocampal modules of the brain depend on artificial neural networks. However, such classical or even deep neural networks are very slow, sometimes taking thousands of trials to obtain the final response with a considerable amount of error. The need for a large number of trials at learning and the inaccurate output responses are due to the complexity of the input cue and the biological processes being simulated. This article proposes a computational model for an intact and a lesioned cortico-hippocampal system using quantum-inspired neural networks. This cortico-hippocampal computational quantum-inspired (CHCQI) model simulates cortical and hippocampal modules by using adaptively updated neural networks entangled with quantum circuits. The proposed model is used to simulate various classical conditioning tasks related to biological processes. The output of the simulated tasks yielded the desired responses quickly and efficiently compared with other computational models, including the recently published Green model.

## Introduction

Several researchers have proposed models that combine artificial neural networks (ANNs) or quantum neural networks (QNNs) with various other ingredients. For example, Haykin (1999) and Bishop (1995) developed multilevel activation function QNNs using the quantum linear superposition feature (Bonnell and Papini, 1997).

The prime factorization algorithm of Shor was used to illustrate the basic workings of QNNs (Shor, 1994). Shor's algorithm uses quantum computations by quantum gates to provide the potential power for quantum computers (Bocharov et al., 2017; Dridi and Alghassi, 2017; Demirci et al., 2018; Jiang et al., 2018). Meanwhile, the work of Kak (1995) focused on the relationship between quantum mechanics principles and ANNs. Kak introduced the first quantum network based on the principles of neural networks, combining quantum computation with convolutional neural networks to produce quantum neural computation (Kak, 1995; Zhou, 2010). Since then, a myriad of QNN models have been proposed, such as those of Zhou (2010) and Schuld et al. (2014).

From 1995 to 2005, many models were developed in the QNN field. This development, and the scientific contributions of the researchers, can be summarized into four main stages. First, researchers attempted to determine the relationship between the nonlinear activation function of neurons in ANNs and the measurement process in quantum mechanics (Schrödinger's famous cat). Second, the concept of logic gates made an appearance in many proposed quantum circuits in the form of quantum gates. Third, researchers specified the requirements and challenges of converting a single-layer ANN to a QNN with the same dynamic features and properties (Schuld et al., 2014). Finally, the first batch of QNNs was introduced, which consisted of interacting quantum dots (qudots); every qudot has two pairs of atoms with two common electrons and interacts with other qudots to form a network (Schuld et al., 2014).

Recently interest in quantum computing has grown rapidly, which has led to increased interest in investigating the applicability of quantum computing to various scientific fields that make use of computational modeling and exhibit certain fundamental characteristics, such as entanglement and superposition. Entanglement is associated with correlated neurons in the ANNs, and linear superposition refers to the linear mathematical relationship. As a result, QNNs have been used in the implementation of an associative memory model (Ventura and Martinez, 2000; Gao et al., 2018), and the US National Aeronautics and Space Administration (NASA) and Google recently utilized QNNs for supervised learning and big data classification in their projects, such as D-Wave processors (Altaisky et al., 2016), and in intelligent controller implementations, such as natural language programming models and robotics (Abdulridha and Hassoun, 2018).

Relatedly, quantum-inspired neural networks (QINNs) (Li and Xiao, 2013) are considered compelling models due to their combination of superposition-based quantum computing and parallel-processed neural computing. QINNs use quantum computation techniques, but these are implemented on classical computers. In other words, a QINN is a classical neural network inspired by quantum computation, just as particle swarm optimization methods are inspired by swarming behavior in organisms such as birds and fish (Kouda et al., 2005). Although there is no experimental evidence that real neurons have specific features in common with QNN models (that are not already included in classical models), adding quantum computing features to artificial neurons can significantly enhance the computational capability of classical neural networks (da Silva and de Oliveira, 2016). The addition of quantum computing features to ANNs has been found to speed up the learning process (Xu et al., 2011; Altaher and Taha, 2017; Lukac et al., 2018). Thus, QINNs have greater computational capability than classical networks (Zhou, 2010). Moreover, the implementation of QINNs has helped us gain better understanding of specific brain functions (Takahashi et al., 2014).

Gluck and Myers (1993) used ANNs to construct a primitive cortico-hippocampal model. The Gluck-Myers model basically consists of two ANNs: an autoencoder and a single-layer feedforward neural network. The autoencoder is an unsupervised neural network that encodes and decodes the input cue to generate internal representations. The single-layer feedforward network is a supervised neural network that processes the input cue along with the internally generated representations to produce the output response, as used in associative learning to simulate basic classical conditioning tasks. These two networks are combined to simulate the intact system of the cortico-hippocampal region. The lesioned system is simulated by severing the link between the supervised and unsupervised networks.

The Gluck-Myers model was improved by Moustafa et al. (2009) to overcome its drawbacks. The Moustafa et al. model is based on more plausible biological paradigms, and uses a feedforward learning algorithm instead of a backpropagation algorithm, which is considered to be an implausible learning rule in biological simulations. Our recent model, the Green model (Khalid et al., 2020), overcomes the weaknesses of the previous two models by using adaptive learning simulated by instar-outstar learning rules.

However, the previously published computational models for simulating cortical and hippocampal modules of the brain (Gluck and Myers, 1993; Moustafa et al., 2009; Khalid et al., 2020) are all based on ANNs. Although the Gluck-Myers and Moustafa et al. models simulated some biological processes using classical neural networks, these models are considered slow and inadequate (Khalid et al., 2020). It is worth mentioning that some classical neural network models do exist that can also learn very fast (by employing one-shot-learning methods), possibly faster than the QINN models proposed in the present paper (Rolls, 1996; Knoblauch et al., 2010).

In this article we propose a cortico-hippocampal computational quantum-inspired (CHCQI) neural network model. Our CHCQI model outperforms the aforementioned models by successfully simulating the same tasks with fewer trials and more plausible output responses. Moreover, the CHCQI model performs better than our previously published Green model (Khalid et al., 2020) in terms of producing a faster response.

## Materials and Methods

### Qubit

**Figure 1:**
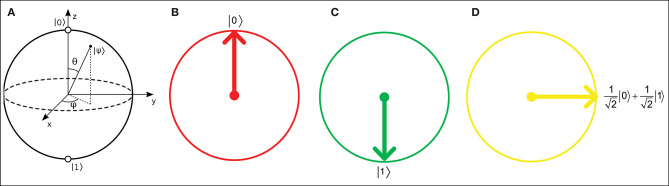
**(A)** Bloch sphere representation of a qubit with real and imaginary probability amplitudes. Real number relative probabilities give the Bloch circle representations for **(B)** a qubit |0〉, **(C)** a qubit |1〉, and **(D)** a 90°-rotated qubit.

A qubit is a quantum mechanical system that is considered the fundamental unit of quantum information. The qubit in quantum computing is regarded as the analog of the bit in classical computing. Unlike the classical bit, which encodes information with two possible states, logic 0 or 1, the qubit uses its properties as a quantum two-level system to encode quantum information as |0〉 and |1〉 states with different probabilities. Accordingly, a qubit state ϕ can have quantum superpositions or a linear combination of the two states as follows:

(1) |ϕ〉=α|0〉+β|1 〉{α,β∈ℝ​},

where α and β are real numbers that represent the relative probabilities of the |0〉 and |1〉 states, respectively. A qubit in a QNN can be visually represented as a Bloch sphere (Bloch, 1946), which has |0〉 and |1〉 states with real and imaginary relative probabilities. However, in QINNs, α and β can each be represented as a slice of a Bloch sphere, i.e., a Bloch circle, as shown in Figure 1. According to Li et al. (2013), a Bloch circle can represent the possible states of any qubit for which the amplitudes of its relative probabilities are real numbers only. Thus, the qubit state can be found in state |0〉 with amplitude probability α and state |1〉 with amplitude probability β, such that

(2) $$\begin{array}{r} |\alpha {|}^{2}+|\beta {|}^{2}=1. \end{array}$$

In a general quantum-inspired system that has *n* qubits with |ϕ_*n*_〉 states, every single state is represented by a unit vector in the Hilbert space. Thus, the general linear superposition of all the unit vector states is expressed as

(3) $$|\psi \rangle =\sum_{n=1}^{N}A_{n}|\phi_{n}\rangle \left\{A_{n}\in \mathbb{R}\text{​}\right\}\;,$$

where |*ψ*〉 is the general quantum state of all the |ϕ_*n*_〉 states and *A*_*n*_ is a real number that represents the probability amplitude of the related state |*n*〉 for 2^*n*^ or *N* states. When the sum of these real number probabilities is equal to 1, as in (2), the sum of the *N* state probabilities is,

(4) $$\begin{array}{r} \underset{n\in {\left\{0,1\right\}}^{n}}{\sum }|A_{n}{|}^{2}=1. \end{array}$$

### Quantum Rotation Gate

In quantum computing, the input-output operations of any circuit can be performed by quantum gates. Quantum gates are similar to logic gates in classical computing but operate with qubits rather than classical bits. We use the Hadamard gate, *H*, which is also known as the Walsh transform, in this study. According to Li et al. (2013), this gate performs qubit superposition by generating equal relative probabilities for every qubit to be either |0〉 or |1〉 as follows:

(5) $$\begin{array}{r} H=\frac{1}{\sqrt{2}}\left[\begin{array}{cc} 1 & 1\\ 1 & -1 \end{array}\right]. \end{array}$$

In other words, the Hadamard gate maps the two states |0〉 and |1〉 together according to the formula

(6) $$H(\alpha |0\rangle +\beta |1\rangle )=\alpha \;(\frac{|0\rangle +|1\rangle }{\sqrt{2}})+\beta \;(\frac{|0\rangle -|1\rangle }{\sqrt{2}}).$$

### Quantum-Inspired Neuron Model

Every node in the CHCQI model has an input-output relation that is represented in the quantum-inspired neuron model as

(7) $$\begin{array}{r} z=f\left(y\right), \end{array}$$

where

(8) $$\begin{array}{r} y=\frac{1}{n_{f}}\overset{n_{f}}{\underset{k}{\sum }}f\left(\delta \left(o-\vartheta^{k}\right)\right), \end{array}$$

with

(9) $$\begin{array}{r} o_{j}=\overset{R}{\underset{i}{\sum }}w_{ij}\cdot f\left(p_{i}\right), \end{array}$$

and

(10) $$\begin{array}{r} f\left(x\right)=\frac{1}{1+e^{-x}}. \end{array}$$

Here *f* is the log-sigmoid activation function, *n*_*f*_ is the number of activation functions, δ is the steepness factor, ϑ represents the quantum intervals, *w*_*ij*_ is the weight matrix of the links from the *i*th node to the *j*th node with *R* qubits, and *p*_*i*_ is the input to the node.

**Figure 2:**
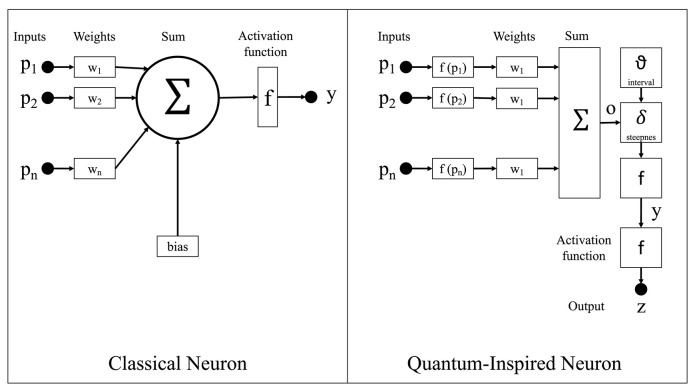
The quantum-inspired neuron model compared with the classical neuron model.

As shown in Figure 2, the classical neuron model in classical neural networks calculates the product of each input to the neuron and its associated weight, sums all these products, and then generates the output via an activation function. On the other hand, the proposed qubit neuron representation in the CHCQI model is the general representation for every node in the network. The calculations follow the same procedure as in a classical network; however, in QINNs the output of a quantum neuron is affected by the values of quantum parameters such as the steepness factor and quantum intervals.

### Proposed Model

**Figure 3:**
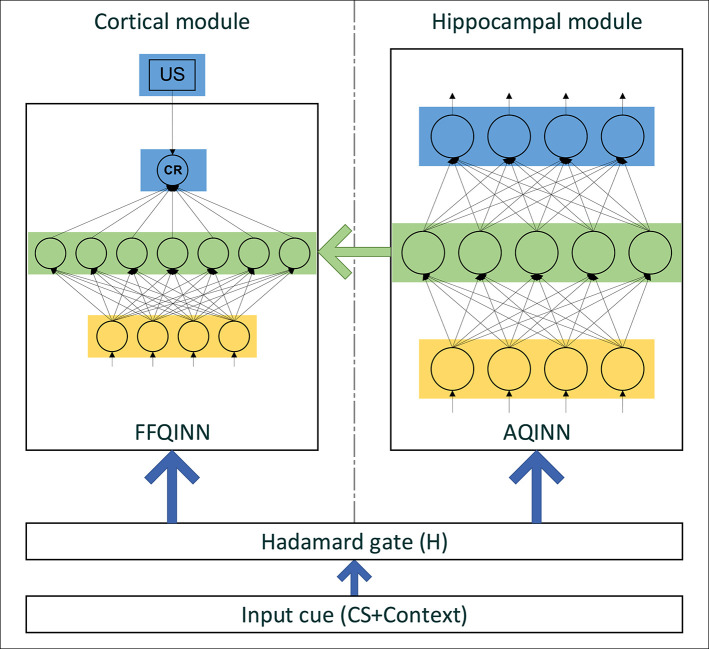
The structure of CHCQI model.

The CHCQI model computationally simulates the cortical and hippocampal modules as shown in Figure 3. The cortical module is represented by a single-hidden-layer feedforward QINN (FFQINN), which updates its weight adaptively depending on the hippocampal module. Meanwhile, the hippocampal module is an autoencoder QINN (AQINN), which encodes the input cue to generate the internal representations. These representations are mapped to the nodes of the hidden layer in the cortical module and are used to update the weights of the hidden layers adaptively.

In the CHCQI model, the intact system comprises cortical and hippocampal modules that connect to the same input from the quantum circuit. The lesioned model has the same structure, but the link that forwards the internal representations from the AQINN to FFQINN is removed, which prevents the adaptive learning.

**Figure 4:**
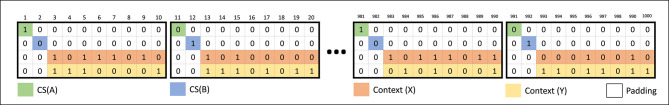
The input data set for the CHCQI model.

Figure 4 illustrates the blocks of input to the CHCQI model. Each input block comprises 10 input cues of binary data, and every input cue consists of four bits. The first bit is assigned to conditioned stimulus (CS) A and the second bit to CS B. The third and fourth bits are assigned to two different contexts, X and Y, with binary values distributed randomly. Wherever one of the conditioned stimuli exists, the other CS and the context values are taken to be zeros, followed by a subsequent zero-padding row. The output response of the network is considered a conditioned response (CR) regarding the unconditioned stimulus (US) target. The CR takes values between 0 and 1. Throughout the learning trials, the value of the output CR changes according to its related task. The learning ends when the CR value reaches a desired unchanging state, which is the steady state.

The biological processes simulated by the CHCQI model are listed in Table 1. Here “A” and “B” refer to the two input conditioned stimuli, while “X” is the context of the cue. The positive and negative signs refer to the CS-US pairing status, with “+” meaning paired and “−” unpaired.

**Table 1:** Tasks simulated by the CHCQI model, where “A” and “B” are the two input conditioned stimuli, “X” is the context of the cue, and the positive and negative signs refer to the CS-US pairing status, with “+” meaning paired and “−” unpaired.

**No**.	**Task name**	**Phase 1**	**Phase 2**	**Phase 3**
1	A+	AX+	—	—
2	A−	AX−	—	—
3	Blocking	AX+	ABX+	BX−
4	Stimulus discrimination	AX+, BX−	—	—
5	Discrimination reversal	AX+, BX−	AX−, BX+	—
6	Easy-hard transfer	A1X+, A2X−	A3X+, A4X−	—
7	Context sensitivity (context shift)	AX+	AY+	—
8	Context sensitivity of latent inhibition	AX−	AY+	—
9	Generic feedforward multilayer network	AX−	AX+	—
10	Sensory preconditioning	ABX−	AX+	BX−
11	Latent inhibition	AX−	AX+	—
12	Overshadowing	ABX+	AX+, BX+	—
13	Compound preconditioning	ABX−	AX+, BX−	—

### Quantum-Input Module

The Hadamard gate initially rotates the phase of each input *p*_*i*_ to obtain a rotated-phase input $\tilde{p_{i}}$. This gate is useful because it can take a qubit into and out of superpositions. If the input is a qubit 0, then |0〉 turns it into a qubit right. Qubit right is halfway between |0〉 and |1〉 simultaneously, as can be seen by rewriting (6) as

(11) $$\tilde{p_{i}}=H(p_{i})=\left(\frac{\alpha +\beta }{\sqrt{2}}\right)|0\rangle +\left(\frac{\alpha -\beta }{\sqrt{2}}\right)|1\rangle .$$

### Hippocampal Module

The weights and quantum circuit parameters in the input layer of the AQINN are updated using the instar learning algorithm, whereas the output layer uses the outstar algorithm to update its weights. The instar and outstar learning algorithms were developed by Grossberg (1967). Typically, these two learning rules encode and decode the input cue to generate internal representations with a plausible error for the updating of network weights (Jain and Chakrawarty, 2017). The updating procedures for the input and output layers are as follows:

(12) $$W_{ij}^{\text{instar}}(n+1)\;=W_{ij}^{\text{instar}}(n)+\mu a_{j}^{h1}(n)\;({\tilde{p^{T}}}_{i}(n)-W_{ij}^{h1}(n)),$$

(13) $$W_{ij}^{\text{outstar}}(n+1)\;=W_{ij}^{\text{outstar}}(n)+\mu (a_{j}^{h2}(n)-W_{ij}^{h2}(n))a_{i}^{h1T}(n),$$

where μ is the learning rate, $a_{j}^{h1}$ is the internal representation output of the AQINN with *Q* qubits, $a_{j}^{h2}$ is the actual output of the AQINN with *R* outputs, $\tilde{p}$ is the rotated-phase input with *R* inputs, the superscript *T* refers to matrix transpose, $W_{ij}^{h1}$ is the internal-layer weight matrix of the links from the *i*th input node to the *j*th hidden qubit of the AQINN with *R* qubits and *Q* hidden nodes, and $W_{ij}^{h2}$ is the weight matrix of the link from the *i*th AQINN hidden qubit to the *j*th corresponding output. Note that (*n*) refers to the current state and (*n* + 1) is the succeeding or new state.

Meanwhile, the quantum interval ϑ^*k*^ is randomly initialized and updated using a gradient descent learning rule as follows:

(14) $$v=(1-f(\delta (o-\vartheta^{k})))\cdot f(\delta (o-\vartheta^{k})),$$

(15) $$\vartheta_{n+1}^{k}=\vartheta_{n}^{k}-\mu \;\left(-\frac{\delta }{n_{f}}(\bar{y}-y)(\bar{v}-v)\right)\text{​},$$

where ȳ and $\bar{v}$ are the normalized vectors of *y* and *v* defined in (8) and (14), respectively.

### Cortical Module

The FFQINN uses two learning algorithms to update its weights, the quantum instar algorithm for the hidden layer and the Widrow-Hoff learning rule for the output layer:

(16) $$W_{ij}^{\text{instar}}(n+1)=W_{ij}^{\text{instar}}(n)+\mu a_{j}^{c1}(n)({\tilde{p}}_{i}^{T}(n)-W_{ij}^{c1}(n)),$$

(17) $$W_{ij}^{c2}(n+1)=W_{ij}^{c2}(n)+\mu a_{i}^{c1}(n)e_{j}(n),$$

(18) $$\text{MAE}=\frac{1}{2}\sum_{j=1}^{\text{iter}}e_{j}=\frac{1}{2}\sum_{j=1}^{\text{iter}}\left(y_{j}-d_{j}\right),$$

where μ is the learning rate, $a_{j}^{c1}$ is the internal representation of the output vector of the FFQINN with *Q* nodes, $\tilde{p_{i}}$ is the rotated-phase input of the FFQINN with *R* inputs, $W_{ij}^{c1}$ is the internal-layer weight matrix of the links from the *i*th input node to the *j*th hidden qubit of the FFQINN with *R* inputs and *Q* qubits, $W_{i,j}^{c2}$ is the upper-layer weight matrix of the FFQINN from input *p*_*i*_ to output node *y*_*j*_, *d*_*j*_ is the desired *j*th output or US, and MAE is the mean absolute error between the actual and desired outputs.

The FFQINN uses (7), (8), (9), and (10) to estimate the output CR, and uses (14) and (15) to update the quantum interval value.

In the intact system, the generated internal representations of the AQINN are fed into the hidden layer of the FFQINN through a fully connected network. This linking network adaptively updates its weights and quantum parameters using the outstar learning algorithm as in (13). In the lesioned system this linking network has been removed.

The AQINN and the FFQINN each have one hidden layer, with 1 and 16 hidden quantum neurons, respectively. Accordingly, the numerical values of the different parameters are estimated as follows: μ of the hippocampal module is 0.03, while μ of the cortical module is 0.01; also, we assume that there is only one activation function, i.e., *n*_*f*_ = 1, and that δ = 1 and ϑ = 2.

## Results

The CHCQI model was used to simulate the tasks in Table 1. The model estimated the number of trials needed to obtain the exact desired output (either 0 or 1) in every task. Most of the tasks comprised two learning phases to complete their simulation, while some tasks had one or three phases.

**Figure 5:**
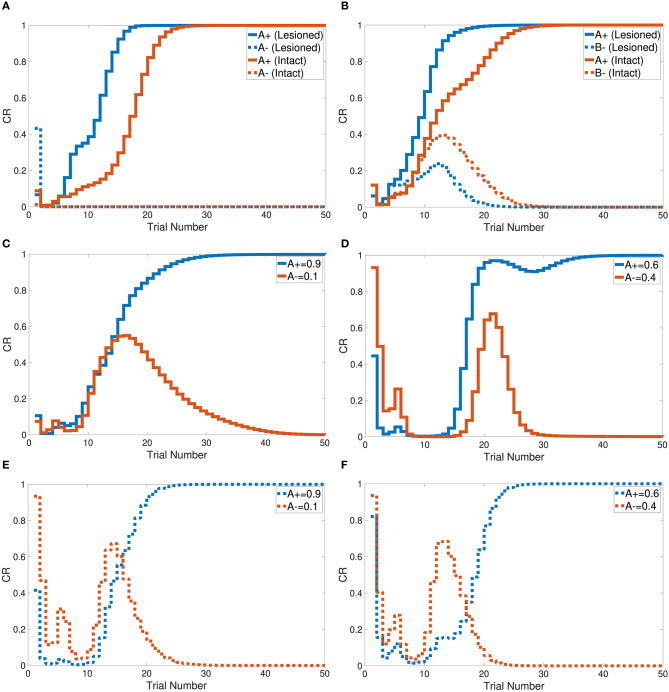
Output responses of the **(A)** A+ and A− learning tasks, **(B)** stimulus discrimination task, **(C)** easy transfer task in the intact system, **(D)** hard transfer task in the intact system, **(E)** easy transfer task in the lesioned system, and **(F)** hard transfer task in the lesioned system.

Tasks 1 and 2 are primitive tasks that were used to demonstrate the basic responses of the intact and lesioned versions of the CHCQI model. The A+ and A− tasks represent the CS-US pairing and unpairing processes by using one input CS in only one phase. Figure 5A shows that the CHCQI model simulated the A+ and A− tasks for the intact and lesioned systems successfully with 26 and 18 trials, respectively. It clearly illustrates the lesioning effect on the CHCQI model, which performed the two tasks in the lesioned system faster than in the intact system. The delayed response of the intact system is due to the forwarding of the generated internal representation from the internal network of the hippocampal module to the internal network of the cortical module adaptively through the learning process. This is the likely cause of every delayed response of the intact system compared with the lesioned system for the rest of the tasks in Table 1.

The CHCQI model was then applied to discriminate two conditioned stimuli within one phase only. The intact and lesioned systems discriminated A+ and B− with a rapid response that reached the final state successfully as shown in Figure 5B. As with tasks 1 and 2, the lesioned system simulated the stimulus discrimination task faster than the intact system, in 19 trials.

**Figure 6:**
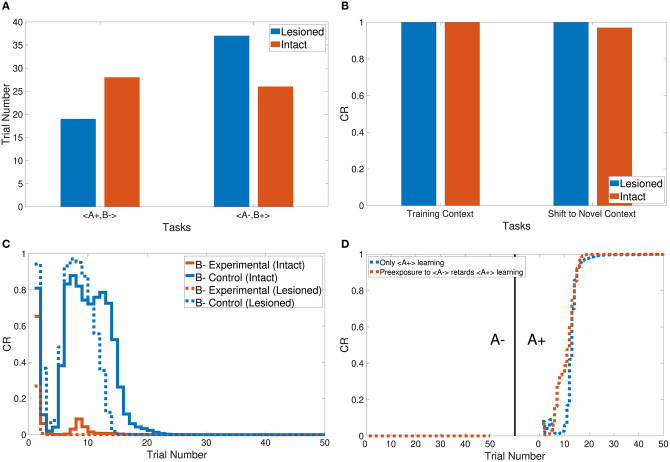
Output responses of the **(A)** discrimination reversal task, **(B)** context sensitivity task, **(C)** blocking task, and **(D)** A+ lesioned system learning task in the CHCQI model compared with the generic feedforward multilayer network.

In addition, the CHCQI model was used to examine the ability of the intact and lesioned systems to discriminate the same two conditioned stimuli after reversing them in a subsequent phase. Unlike in the first phase, the lesioned system needed more trials than the intact system to discriminate the two stimuli at the second phase, as shown in Figure 6A; the lesioned system took 37 trials, approximately double the number of trials in the first phase, while the intact system showed only a slight increase, of two more trials, relative to the first phase. This increased number of trials in the lesioned system can be explained by the cortical module receiving no internal representations forwarded by the hippocampal module, whereas the intact system received internal representations from the hippocampal side that had been trained during the first phase.

Task 3 examines the blocking and unblocking effects of one CS on another by using two stimuli due to a prior conditioning phase. The third phase is the control CS, which can be blocked by adding the response from the preceding phase to get the experimental CS. As a result, the experimental B− in the third phase obtained its response from the second phase. However, B− was blocked by A+ in the first phase. The intact system displayed the blocking effect, while the lesioned system was not affected by the blocking, as shown in Figure 6C.

**Figure 7:**
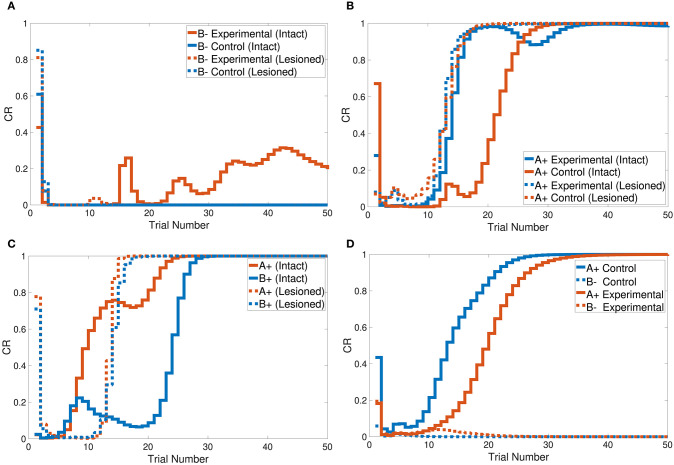
Output responses of the **(A)** sensory preconditioning task, **(B)** latent inhibition task, **(C)** overshadowing task, and **(D)** compound preconditioning task.

The CHCQI model simulated the overshadowing task in two phases. In the first phase two conditioned stimuli were learned together (AB+), and in the second phase these two stimuli were learned simultaneously (A+, B+). The intact system's response showed overshadowing of B+ by A+, with both subsequently reaching their final states as shown in Figure 7C. The lesioned system eliminated the overshadowing effect of A+ on B+, which attained their final states together at around the same time.

Task 6 involves stimulus discrimination with different amplitude values of the CS. The easy transfer task was simulated with A+ and A− equal to 0.9 and 0.1, respectively; the hard transfer task reduced the gap between A+ and A−, which were taken to be 0.6 and 0.4, respectively. Similar to tasks 1, 2, and 3, the CHCQI model simulated the easy and hard transfer tasks successfully for the intact and lesioned systems. The lesioned system took 24 trials for the easy transfer task and 26 for the hard transfer task, as shown in Figures 5C–F. The intact system took 30 and 37 trials for the easy transfer and hard transfer tasks, respectively.

Figure 7B shows that the intact system produced its response to the latent inhibition task after 35 trials. The successful delayed response of the intact system is due to the generated representation at the pre-exposure phase. The lesioned system, on the other hand, did not show any response to the latent inhibition task because of the lack of forwarded representations from the hippocampal module.

Tasks 10 and 13 investigate the effects of related context on CS-US conditioning in different situations. The intact system successfully simulated the sensory and compound preconditioning tasks within three and two phases, respectively. Figure 7A shows the third-phase response of the sensory preconditioning task, and Figure 7D shows the second-phase response of the compound preconditioning task. It is clearly seen that the discrimination response for the experimental CS A and CS B took more trials than for the control stimuli.

The CHCQI model simulated the context sensitivity task in the intact system by shifting the context of the relevant CS during the second phase. Figure 6B shows the context shifting effect in the intact system with and without the latent inhibition effect, by comparing the response values of the last and first trials of the first and second phases, respectively.

Moreover, Figure 6D shows the response of the lesioned system to A+ learning after the pre-exposure phase, demonstrating the similarity of the lesioned system's response to the supervised feedforward neural network response proposed by Rumelhart et al. (1986).

## Discussion

The CHCQI model simulated all the biological process tasks listed in Table 1 and estimated the number of trials needed to obtain the exact desired output (either 0 or 1) in each task. Most of the tasks required two learning phases to complete their simulation, whereas some tasks comprised one phase or three phases.

The first two tasks are the CS-US pairing and unpairing tasks using one input CS. Tasks 3 and 10 examined the blocking and unblocking effects, respectively, of one CS on another due to a prior conditioning phase. Tasks 4 and 5 pertain to the discrimination of two conditioned stimuli and their reversed pair. Tasks 6, 7, 8, 9, and 11 investigated the effects of the related context on the CS-US conditioning in different situations. Tasks 12 and 13 examined overshadowing and compound preconditioning effects during simulation with two different conditioned stimuli simultaneously according to the preconditioning phase.

The CHCQI model successfully produced the desired outputs of all the simulated tasks with a consistent CR for intact and lesioned systems. All the output CRs reached their final states (either 0 or 1) directly after a plausible number of training trials, as shown in Figures 5–7. Moreover, in nearly all the tasks, the lesioned system completed the learning faster (i.e., with a fewer number of trials) than the intact system. By contrast, the discrimination reversal task took several more trials in the lesioned system. Although the lesioned system learned faster than the intact system, it has no link with the adaptively generated internal representations. Thus, the lesioned system took a long time to re-discriminate a reversed pair of conditioned stimuli as new input.

**Figure 8:**
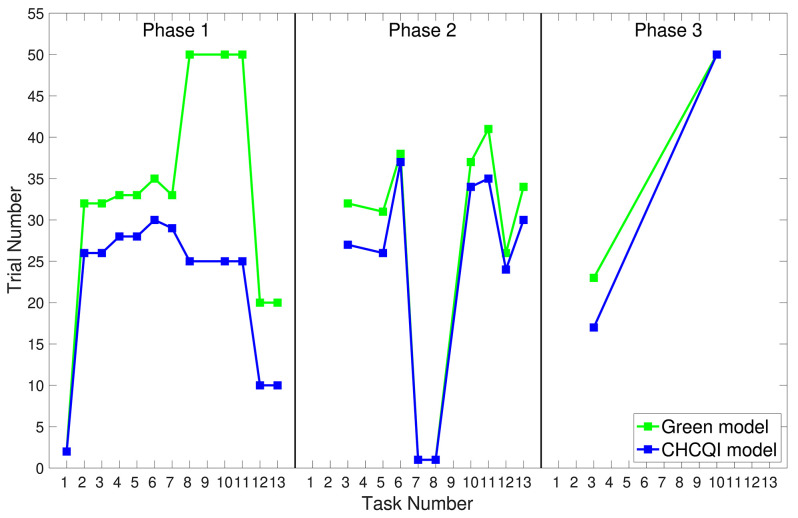
Comparison of the three phases for the intact system in the CHCQI and Green models.

**Figure 9:**
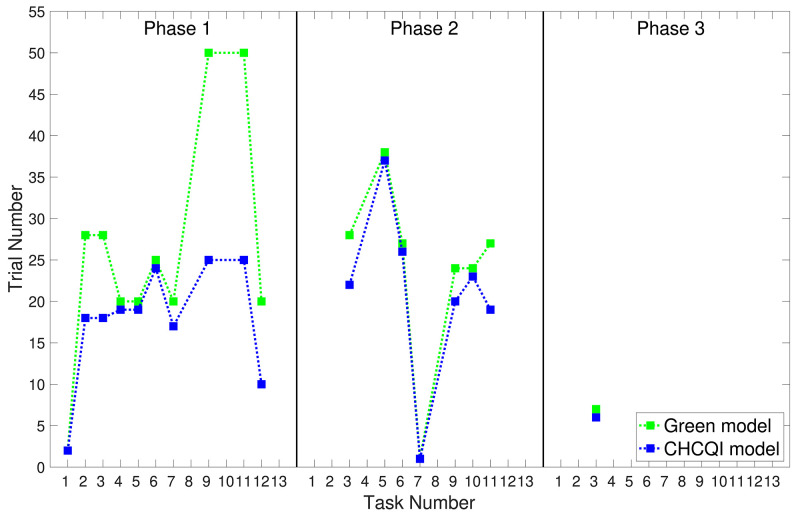
Comparison of the three phases for the lesioned system in the CHCQI and Green models.

As with the Green model (Khalid et al., 2020), in all the simulated tasks the CHCQI model yielded results consistent with the aforementioned experimental studies. However, the CHCQI model produced output CRs faster than the Green model for all the three-phase tasks in both the intact and the lesioned systems as shown in Table 2. Figures 8, 9 plot the number of trials taken to obtain the desired output in each task. The results indicate that the CHCQI model is an improvement over the Green model in the sense of obtaining the output CR faster in most tasks in the three phases.

**Table 2:** Comparison of the Green and CHCQI models in terms of the number of trials needed to attain the final state of the CR.

**No**.	**Task name**	**Phase 1**						**Phase 2**						**Phase 3**					
		**(a)**	**(b)**	**(c)**	**(d)**	**Improvement**		**(e)**	**(f)**	**(g)**	**(h)**	**Improvement**		**(i)**	**(j)**	**(k)**	**(l)**	**Improvement**	
		**G_I_**	**CHCQI_I_**	**G_L_**	**CHCQI_L_**	**(b) vs. (a)**	**(d) vs. (c)**	**G_I_**	**CHCQI_I_**	**G_L_**	**CHCQI_L_**	**(f) vs. (e)**	**(h) vs. (g)**	**G_I_**	**CHCQI_I_**	**G_L_**	**CHCQI_L_**	**(j) vs. (i)**	**(l) vs. (k)**
1	A−	2	**2**	2	**2**	00.0%	00.0%	—	—	—	—	—	—	—	—	—	—	—	—
2	A+	32	**26**	28	**18**	18.8%	35.7%	—	—	—	—	—	—	—	—	—	—	—	—
3	Blocking	32	**26**	28	**18**	18.8%	35.7%	32	**27**	28	**22**	15.6%	21.4%	23	**17**	7	**6**	26.1%	14.3%
4	Stimulus discrimination	33	**28**	20	**19**	15.2%	05.0%	—	—	—	—	—	—	—	—	—	—	—	—
5	Discrimination reversal	33	**28**	20	**19**	15.2%	05.0%	31	**26**	38	**37**	16.1%	02.6%	—	—	—	—	—	—
6	Easy-hard transfer	35	**30**	25	**24**	14.3%	04.0%	38	**37**	27	**26**	02.6%	03.7%	—	—	—	—	—	—
7	Context sensitivity	33	**29**	20	**17**	12.1%	15.0%	1	1	1	1	00.0%	00.0%	—	—	—	—	—	—
8	Context sensitivity of latent inhibition	50	**25**	—	—	50.0%	—	1	1	—	—	00.0%	—	—	—	—	—	—	—
9	Generic feedforward multilayer network	—	—	50	**25**	—	50.0%	—	—	24	**20**	—	16.7%	—	—	—	—	—	—
10	Sensory preconditioning	50	**25**	—	—	50.0%	—	37	**34**	—	—	08.1%	—	50	50	—	—	00.0%	—
11	Latent inhibition	50	**25**	50	**25**	50.0%	50.0%	41	**35**	24	**23**	14.6%	04.2%	—	—	—	—	—	—
12	Overshadowing	20	**10**	20	**10**	50.0%	50.0%	26	**24**	27	**19**	07.7%	29.6%	—	—	—	—	—	—
13	Compound preconditioning	20	**10**	—	—	50.0%	—	34	**30**	—	—	11.8%	—	—	—	—	—	—	—

*Bold emphasis indicates significant variance in the numbers of trials*.

**Figure 10:**
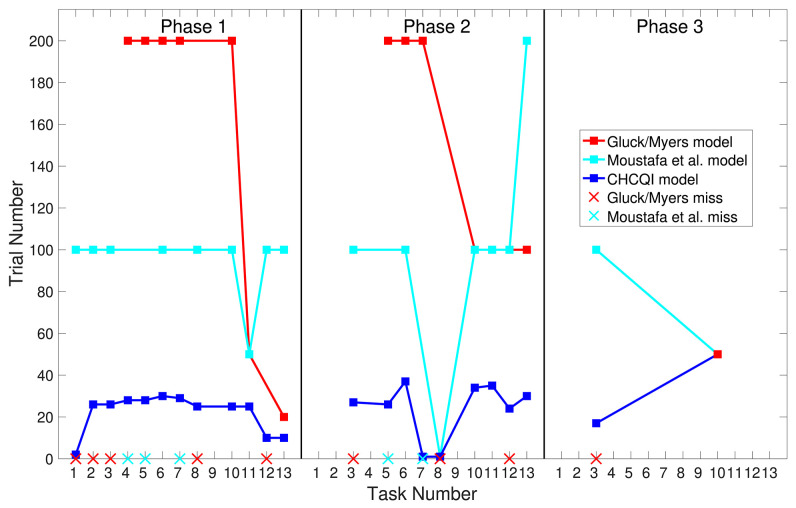
Comparison of the three phases for the intact system in the CHCQI, Gluck-Myers, and Moustafa et al. models. The red and cyan crosses represent missed tasks that were not simulated by the Gluck-Myers and Moustafa et al. models. For better visualization of the comparison, the number of trials for task 6 in the second phase of the Gluck-Myers model has been scaled to 200 from 1,000.

**Figure 11:**
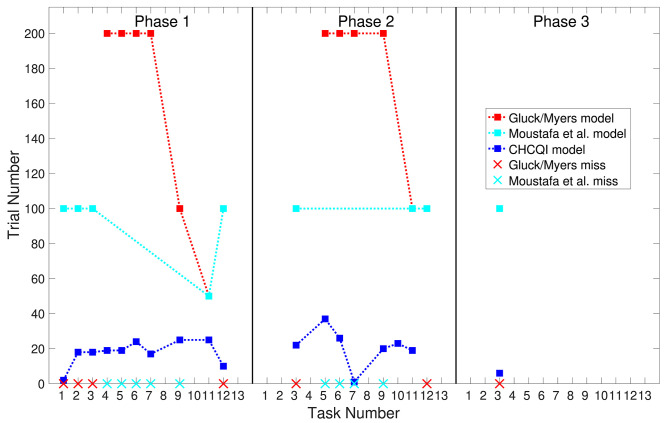
Comparison of the three phases for the lesioned system in the CHCQI, Gluck-Myers, and Moustafa et al. models. The red and cyan crosses represent missed tasks that were not simulated by Gluck-Myers and Moustafa et al. models. For better visualization of the comparison, the numbers of trials for tasks 5 and 6 in the second phase of the Gluck-Myers model have been scaled to 200 from 400 and 1,000, respectively.

Also, Tables 3, 4 show the number of trials needed to attain the final state of the CRs compared with the Gluck-Myers and Moustafa et al. models respectively. The CHCQI model also obtains output CRs more rapidly, as shown in Figures 10, 11. Unlike the CHCQI model, the Gluck-Myers and Moustafa et al. models are incapable of simulating several tasks.

**Table 3:** Comparison of the Gluck-Myers and CHCQI models in terms of the number of trials needed to attain the final state of the CR.

**No**.	**Task name**	**Phase 1**						**Phase 2**						**Phase 3**					
		**(a)**	**(b)**	**(c)**	**(d)**	**Improvement**		**(e)**	**(f)**	**(g)**	**(h)**	**Improvement**		**(i)**	**(j)**	**(k)**	**(l)**	**Improvement**	
		**M1_I_**	**CHCQI_I_**	**M1_L_**	**CHCQI_L_**	**(b) vs. (a)**	**(d) vs. (c)**	**M1_I_**	**CHCQI_I_**	**M1_L_**	**CHCQI_L_**	**(f) vs. (e)**	**(h) vs. (g)**	**M1_I_**	**CHCQI_I_**	**M1_L_**	**CHCQI_L_**	**(j) vs. (i)**	**(l) vs. (k)**
4	Stimulus discrimination	>200	**28**	>200	**19**	86.0%	90.5%	—	—	—		—	—	—	—	—	—	—	—
5	Discrimination reversal	>200	**28**	>200	**19**	86.0%	90.5%	>200	**26**	>400	**37**	87.0%	90.8%	—	—	—	—	—	—
6	Easy-hard transfer learning	>200	**30**	>200	**24**	80.0%	88.0%	>1000	**37**	>1000	**26**	96.3%	97.4%	—	—	—	—	—	—
7	Context sensitivity	>200	**29**	>200	**17**	85.5%	91.5%	>200	1	>200	1	99.5%	99.5%	—	—	—	—	—	—
9	Generic feedforward multilayer network	—	—	100	**25**	—	75.0%	—	—	>200	**20**	—	90.0%	—	—	—	—	—	—
10	Sensory preconditioning	200	**25**	—	—	87.5%	—	>100	**34**	—	—	66.0%	—	50	50	—	—	00.0%	—
11	Latent inhibition	50	**25**	50	**25**	50.0%	50.0%	>100	**35**	>100	**23**	65.0%	77.0%	—	—	—	—	—	—
13	Compound preconditioning	20	**10**	—	—	50.0%	—	>100	**30**	—	—	70.0%	—	—	—	—	—	—	—

*Bold emphasis indicates significant variance in the numbers of trials*.

**Table 4:** Comparison of the Moustafa et al. and CHCQI models in terms of the number of trials needed to attain the final state of the CR.

**No**.	**Task name**	**Phase 1**						**Phase 2**						**Phase 3**					
		**(a)**	**(b)**	**(c)**	**(d)**	**Improvement**		**(e)**	**(f)**	**(g)**	**(h)**	**Improvement**		**(i)**	**(j)**	**(k)**	**(l)**	**Improvement**	
		**M2_I_**	**CHCQI_I_**	**M2_L_**	**CHCQI_L_**	**(b) vs. (a)**	**(d) vs. (c)**	**M2_I_**	**CHCQI_I_**	**M2_L_**	**CHCQI_L_**	**(f) vs. (e)**	**(h) vs. (g)**	**M2_I_**	**CHCQI_I_**	**M2_L_**	**CHCQI_L_**	**(j) vs. (i)**	**(l) vs. (k)**
1	A−	>100	**2**	>100	**2**	98.0%	98.0%	—	—	—	—	—	—	—	—	—	—	—	—
2	A+	>100	**26**	>100	**18**	74.0%	82.0%	—	—	—	—	—	—	—	—	—	—	—	—
3	Blocking	>100	**26**	>100	**18**	74.0%	82.0%	>100	**27**	>100	**22**	73.0%	78.0%	>100	**12**	>100	**3**	88.0%	97.0%
6	Easy-hard transfer learning	>100	**30**	—	—	70.0%	—	>100	**37**	—	—	63.0%	—	—	—	—	—	—	—
7	Context shift	100	**25**	—	—	75.0%	—	1	1	—	—	00.0%	—	—	—	—	—	—	—
8	Context sensitivity of latent inhibition	100	**25**	—	—	75.0%	—	1	1	—	—	00.0%	—	—	—	—	—	—	—
10	Sensory preconditioning	100	**25**	—	—	75.0%	—	>100	**34**	—	—	66.0%	—	50	50	—	—	00.0%	—
11	Latent inhibition	50	**25**	50	**25**	50.0%	50.0%	>100	**35**	>100	**23**	65.0%	77.0%	—	—	—	—	—	—
12	Overshadowing	100	**10**	100	**10**	90.0%	90.0%	>100	**24**	>100	**19**	76.0%	81.0%	—	—	—	—	—	—
13	Compound preconditioning	100	**10**	—	—	90.0%	—	>200	**30**	—	—	85.0%	—	—	—	—	—	—	—

*Bold emphasis indicates significant variance in the numbers of trials*.

Finally, comparing the CHCQI model, which uses QINNs, with models that use spiking neural networks (SNNs) to simulate cortico-hippocampal regions, the CHCQI model relies mainly on the Widrow-Hoff, instar, and outstar learning rules to adaptively update the weights of the networks according to the internally generated representations, whereas the SNN-based models depend on the adapted spiking time. Thus, updating the weights of the network is processed by the phase of the theta oscillation that is generated externally by a spiking modulator (Tielin et al., 2016; Parish et al., 2018; Zhao et al., 2018).

## Conclusions

This article has proposed the CHCQI model for cortico-hippocampal regions, which outperforms several previously published models, such as the Gluck-Myers, Moustafa et al., and Green models. Across a variety of simulated tasks, the CHCQI model shows fast responses, requiring fewer trials to reach the desired final states. In addition, the CHCQI model is capable of simulating more biological processes than other models. QINNs are used to build the CHCQI model because quantum circuits afford computational speedup over ANNs for certain multivariate problems. The powerful parallel-computing aspect enables even better performance of QINNs than ANNs.

The use of quantum computation in the CHCQI model makes the model more powerful at simulating the cortico-hippocampal region than classical computational models. Instead of simulating an *n*-bit information input cue as in classical computation, a quantum computation simulates the same cue as 2^*n*^ possible states. The quantum circuit (including quantum rotation gates) allows computations to be speeded up relative to ANNs in classical conditioning simulations. Such accelerated computation enables the CHCQI model to simulate many biological paradigms efficiently.

## Data Availability Statement

The raw data supporting the conclusions of this article will be made available by the authors, without undue reservation.

## Author Contributions

MK and JW conceived of the presented idea. MK developed the theory and simulated the computations. AM verified the biological processes results. TMA, TA, and AA encouraged MK to investigate quantum theory and quantum computations. JW, QZ, and RX supervised the findings of this work. All authors discussed the results and contributed to the final manuscript.

## Conflict of Interest

The authors declare that the research was conducted in the absence of any commercial or financial relationships that could be construed as a potential conflict of interest.
